# Lp(a): When and how to measure it

**DOI:** 10.1177/0004563220968473

**Published:** 2020-10-28

**Authors:** Jaimini Cegla, Michael France, Santica M Marcovina, R Dermot G Neely

**Affiliations:** 1 Division of Diabetes, Endocrinology and Metabolism, Imperial College London, London, UK; 2 Department of Clinical Biochemistry, Manchester University NHS Foundation Trust, Manchester, UK; 3 Medpace Reference Laboratories, Cincinnati, OH, USA; 4 Department of Blood Sciences and NIHR MedTech and IVD Centre, Newcastle Upon Tyne Hospitals, Newcastle Upon Tyne, UK

**Keywords:** Lp(a), lipoprotein(a), measurement of Lp(a), measurement of lipoprotein(a)

## Abstract

Lipoprotein(a) has long been regarded as a risk factor for cardiovascular disease; however, its routine use in clinical practice has been hampered by difficulties inherent in the measurement of this complex lipoprotein. The major challenges relate to its size heterogeneity and related issues including (1) use of appropriate calibrators (2) standardization of calibration protocols (3) traceability and (4) reporting units. In the UK, results from the current EQA schemes for lipoprotein(a) suggest that there is considerable work required to standardize lipoprotein(a) measurement. This is becoming increasingly pertinent with the increasing recognition of lipoprotein(a) as an independent risk factor for cardiovascular disease in international guidelines and the emergence of novel antisense therapies to effectively reduce lipoprotein(a). This article raises awareness of the importance of measurement of lipoprotein(a) for the assessment of cardiovascular disease risk and gives guidance to clinical laboratories regarding choice of appropriate assays.

## Why should Lp(a) be measured?

Lipoprotein(a) (Lp(a)) has long been regarded as a risk factor for cardiovascular disease (CVD); however, it is only in recent years that its causal role in CVD has fully been appreciated.^1^ First described by Kåre Berg in 1963 as an antigenically distinct form of beta-lipoprotein, Lp(a) was detected by immunoprecipitation techniques in approximately one-third of individuals with an apparent autosomal co-dominant mode of inheritance.^2^ The greater significance of Lp(a) as a genetic risk factor for coronary heart disease was not appreciated until more than a decade later when it was found to be synonymous with the pre-beta 1 and ‘sinking’ pre-beta lipoprotein (SBPL) bands in agarose gels.^3^ The presence of SBPL was associated with increased risk of premature myocardial infarction and higher concentration of Lp(a) as measured by a newly developed quantitative immunoassay method.^4^ However, evidence from prospective studies was conflicting, results from those using immunoassays being inconsistent^5^,^6^ but those reporting presence of SBPL consistently supporting higher Lp(a) concentration as a CHD risk factor.^7^,^8^ Only in more recent years, with advances in genetics and the application of Mendelian randomization analysis in large population studies, has the role of Lp(a) as a causal cardiovascular risk factor been proven beyond doubt.^1^,^9^

**Figure 1.**
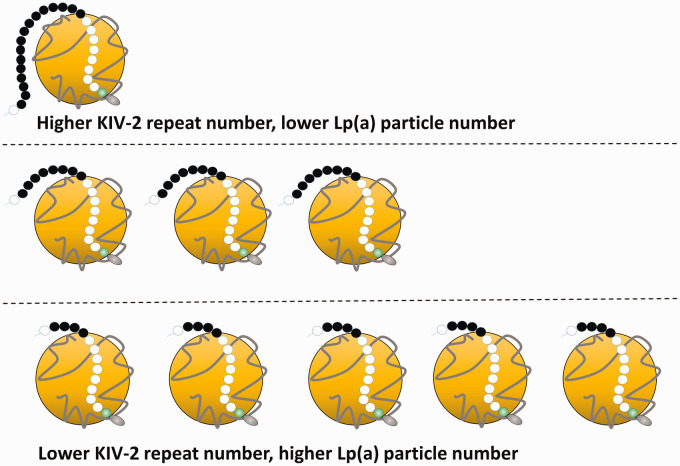
(a) Lp(a) is an LDL-like particle containing apoB (grey ribbon) bound to apo(a) (black and white circles). The black circles represent the Kringle IV-2 domain, with variable number of repeats apo(a). Lp(a) plasma concentrations are inversely proportional to the number of Kringle IV type 2 repeats on apo(a).

Composed of an LDL-like particle, in which a single apolipoprotein B100 (apoB) is covalently linked by a disulphide bond to a single apolipoprotein(a) (apo(a)) (Figure 1), Lp(a) presents several unique challenges as a measurand. Among individuals, the molecular weight of apo(a) can vary between 275 and 800 kDa. This is due to the possibility of inheriting one of >40 different isoforms of apo(a),^10^ largely defined by the number of repeats of the kringle IV type 2 coded sequence, which may range from fewer than 3 to greater than 40 in number.^11^ In addition, the rate of synthesis of apo(a) in hepatocytes is inversely correlated to apo(a) size and consequently, those with lower molecular weight apo(a) isoforms generally have higher plasma concentrations of Lp(a). Remarkably, Lp(a) plasma concentrations within the population can therefore vary by 1000-fold. The pathophysiology underpinning Lp(a)’s role in atherosclerosis is thought to be two-fold:^12^,^13^ firstly, a pro-thrombotic effect by inhibiting fibrinolysis due to the sequence homology of apo(a) to plasminogen and secondly, a pro-atherogenic effect through its ability to accumulate in the intima of arteries. Epidemiological and genetic data have now confirmed Lp(a) as an independent risk factor for coronary heart disease, stroke and calcific aortic valve stenosis.^14–16^

## When should Lp(a) be measured?

HEART UK (Hyperlipidaemia Education and Atherosclerosis Research Trust UK) recently published a consensus statement that makes recommendations regarding the use of Lp(a) measurement in clinical practice and also reviews current and emerging therapeutic strategies to reduce plasma Lp(a) concentrations to decrease risk of CVD. HEART UK recommends Lp(a) measurement in the following groups:

- A personal or family history of premature atherosclerotic cardiovascular disease (<60 years of age).
- First degree relatives of those with high Lp(a) plasma concentrations (>200 nmol/L).
- Familial hypercholesterolaemia (FH), or other genetic forms of dyslipidaemia.
- Calcific aortic valve stenosis.
- A borderline increased (but <15%) 10-year risk of a cardiovascular event.

As plasma concentrations of Lp(a) are predominantly genetically determined, they are relatively stable over a lifetime. Therefore, Lp(a) may only need to be measured once, unless a secondary cause is suspected or a specific treatment is instituted in order to lower its plasma concentration. The cardiovascular risk conferred by Lp(a) may be graded depending on the lipoprotein(a) particle concentration and HEART UK have employed data from the ongoing Copenhagen General Population Study^17^ to grade this risk on the basis of percentile distributions as follows: 32–90 nmol/L minor; 90–200 nmol/L moderate; 200–400 nmol/L high; >400 nmol/L very high. Further studies are required to derive ethnicity-specific ranges, appropriate to the UK population as Lp(a) concentration varies by race/ethnicity.^18^

## How should Lp(a) be measured?

Despite the efforts of the International Federation of Clinical Chemistry (IFCC) to develop a reference material for standardization of analytical methods (WHO/IFCC SRM-2B), shortcomings of commercially available immunoassays for this complex particle, primarily due to their sensitivity to apo(a) isoform size variation, have long been apparent^19^ and over the past two decades the questionable performance of these assays has hampered the incorporation of Lp(a) measurement into routine clinical practice.^20^ However, the recent demonstration that the genetic risk of variants in the *LPA* gene is fully captured by measurement of Lp(a) particle numbers (expressed in nmol/L) using commercial immunoassays standardized and calibrated to minimize isoform sensitivity^21^ shows that reliable methods for assessment of Lipoprotein(a) associated CVD risk are available on modern high throughput platforms. There is now an urgent need for assay harmonization to allow reliable clinical decision making.

The large heterogeneity in apo(a) size between, as well as within individuals because of the inheritance of two different apo(a) alleles, has been a major challenge to the accurate measurement of Lp(a).^22^ The antibodies used against apo(a) are usually polyclonal and cross-react with the multiple KIV-2 repeats thereby overestimating Lp(a) plasma concentrations in individuals with large isoforms and underestimating the concentrations in those with small isoforms.^22^ As a consequence of this bias, the strength of the relationship between Lp(a) and CVD risk has appeared inconsistent and has frequently been underestimated. More recent studies using a monoclonal antibody-based ELISA^22^ assay insensitive to apo(a) isoform size variability show a consistent positive relationship between high levels of Lp(a) and CVD, whereas earlier studies using isoform sensitive assays have missed this important relationship. In particular, two influential epidemiological studies (the Framingham Study and the Physicians’ Health Study) which reported negative results, were repeated using the isoform insensitive ELISA assay and were clearly positive.^23^ This highlights the importance of using suitable and standardized methods for Lp(a) measurement to accurately assess CVD risk.

Many studies have shown differences between Lp(a) assays.^10^,^11^ Concerted effort was made by the IFCC to select and characterize a suitable reference material and to develop a multistep standardization protocol to be used by manufacturers and clinical laboratories. However, traceability to the WHO/IFCC SRM-2B improves assay comparability but does not eliminate the isoform sensitivity of the analytical methods.^24^ The target value assigned to the reference material is in nanomoles per litre of Lp(a) protein, reflecting a mole for mole interaction of antibody with apo(a). Lp(a) has historically been expressed in mass units (mg/dL) encompassing the mass of the entire particle, including the content of apo(a), apoB-100, cholesterol, cholesteryl ester, phospholipid, triglyceride and carbohydrate. This is metrologically unsound because what is measured by immunoassays is the protein component of Lp(a) and not its lipid and carbohydrate content. Therefore, the most appropriate units of measurement of Lp(a) are nmol/L. Lp(a) concentrations should not be converted from nmol/L to mg/dL, or vice versa, as all conversion factors are inherently isoform dependent.^25^

**Figure 2.**
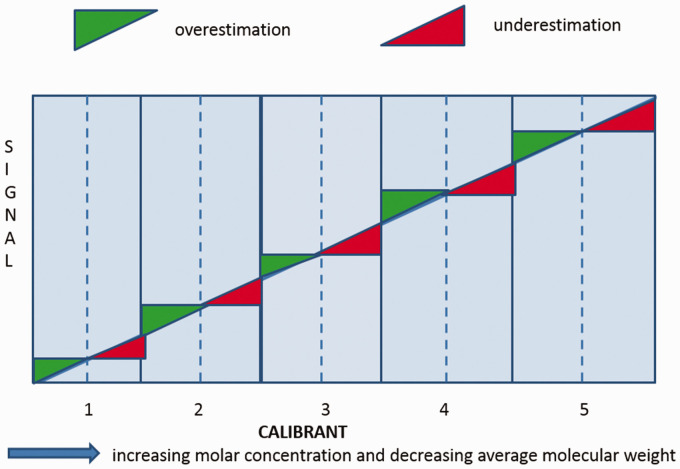
Schematic of the Denka calibration. Calibrators are prepared in separate pools with different levels of Lp(a), and therefore the isoform composition better matches the test sample leading to improved isoform insensitivity.

Let us be very clear. None of the current commercially available assays for Lp(a) measurement are inherently isoform insensitive.^26–28^ Of the commercially available methods (Figure 2), the Denka-based assays (Denka Seiken Co. Ltd, Japan) are currently the least isoform sensitive, primarily due to the use of five calibrators to cover the measured range of concentrations, each calibrator being independent and containing a suitable distribution of apo(a) isoforms, traceable in nmol/L to the WHO/IFCC reference material. A certification process run by the Northwest Lipid Research Laboratory, University of Washington, Seattle is available for evaluating the performance of the different assays by comparing the Lp(a) values with those obtained by the monoclonal antibody-based ELISA method. The list of manufacturers and laboratories is provided in Table 1 with the indication of the certified instrument as well as the date of the last certification.

**Table 1.** List of manufacturers and relative instruments certified by the Northwest Research Lipid Laboratory, University of Washington, Seattle for values being traceable to the WHO/IFCC Reference Material for Lp(a).

Company	Location	Instrument	Last evaluation Date
CTSU Wolfson Laboratories	United Kingdom	Beckman Coulter AU 680	18 August 2015
Denka Seiken Co Ltd	Japan	Olympus AU460	19 June 2012
Denka Seiken Co Ltd	Japan	Beckman AU5800	29 March 2016
Denka Seiken Co Ltd	Japan	Roche Hitachi 917	21 February 2020
DiaSys Diagnostic Systems GmbH	Germany	Roche Hitachi 917	10 July 2018
Diazyme Laboratories	California	Olympus AU400	10 December 2014
MedTest DX	Michigan	Roche Hitachi Modular P	18 October 2016
MedTest DX	Michigan	Roche Cobas c501	24 April 2020
Randox Laboratories	United Kingdom	Abbott Allinity c	29 January 2020
Randox Laboratories	United Kingdom	Abbott Architect C8000	05 July 2013
Randox Laboratories	United Kingdom	Beckman Coulter AU640	11 July 2013
Randox Laboratories	United Kingdom	Randox Binding Site, SPAplus	01 December 2013
Randox Laboratories	United Kingdom	Randox Daytona+	26 September 2019
Randox Laboratories	United Kingdom	Randox Imola	9 August 2019
Randox Laboratories	United Kingdom	Roche Cobas c501	19 February 2020
Randox Laboratories	United Kingdom	Roche Hitachi 717	8 August 2013
Randox Laboratories	United Kingdom	Siemens Atellica CH	4 February 2020
Randox Laboratories	United Kingdom	Siemens Advia 1800	8 November 2013
Randox Laboratories	United Kingdom	Siemens Advia 2400	08 November 2013
Roche Diagnostics GmbH	Germany	Roche Cobas c311	28 August 2017
Roche Diagnostics GmbH	Germany	Roche Cobas c501	28 August 2017
Roche Diagnostics GmbH	Germany	Roche Cobas c503	28 August 2017
Roche Diagnostics GmbH	Germany	Roche Cobas c701	28 August 2017
Roche Diagnostics GmbH	Germany	Roche Cobas Integra 400 Plus	28 August 2017
Roche Diagnostics GmbH	Germany	Roche Cobas Integra 800	28 August 2017
Roche Diagnostics GmbH	Germany	Roche Modular P	28 August 2017
Sentinel CH SpA	Italy	Beckman AU680	5 June 2020
Sentinel CH SpA	Italy	Beckman AU5800	29 May 2020
Shenzhen Mindray Bio-Medical Electronics Co Ltd	China	Mindray BS-800	11 June 2018
UK Biobank	United Kingdom	Beckman AU5822	30 May 2017
UK Biobank	United Kingdom	Beckman AU5800	30 January 2017

We suggest the following to improve harmonization of Lp(a) assays:

- Check that the accuracy of the assay has been certified by the Northwest Lipid Research Laboratory in Seattle making sure the certification applies to the specific instrument.
- Do not convert results to mass units.
- External quality assurance programs should distribute samples with known apo(a) isoform composition and Lp(a) values assigned by a method validated to be independent of apo(a) size polymorphism and calibration traceable to the WHO/IFCC SRM-2B reference material.
- External quality assurance samples should cover the clinically meaningful range, especially the management threshold range of 90–200 nmol/L

## Clinical laboratories and Lp(a) measurement

In the UK, there is a long way to go with assay harmonization. The vast majority of laboratories report Lp(a) concentration in mass units (Table 2), with no traceability to the WHO/IFCC reference assay. For those using Denka-based reagents, laboratory providers need to be confident that their assays employ calibrators traceable in nmol/L to the WHO/IFCC reference material. As discussed, arbitrary conversion from mass to molar units is not acceptable. Clinical laboratories should no longer offer assays without evidence of traceability and assays reported in mass units should be red flagged.

**Table 2.** EQA Schemes and methods for Lp(a) measurement registered in the UK.

Scheme	Method	No of UK labs	Units
WEQAS	Abbott alinity	1	g/L
	Roche cobas^a^	3	g/L
NEQAS	Randox reagents^a^	7	mg/L
	Roche cobas^a^	5	nmol/L
	Roche cobas^a^	1	mg/L
	Beckman (Olympus)	1	mg/L
	Abbott architect	3	mg/L
RIQAS^b^	Randox^a^	6	mg/dL
	Roche cobas^a^	1	mg/dL
	Abbott	1	mg/L
Total		29	

Note: Data kindly provided by WEQAS, NEQAS and RIQAS.

^a^Denka reagents but calibration protocol not disclosed.

^b^Results reported by labs in mg/dL and then converted to nmol/L to calculate the consensus mean (mg/dL multiplied by 2.27 to convert to nmol/L for all methods).

There is clearly a need for innovation in the development of isoform insensitive Lp(a) assays. One approach could be a sandwich immunoassay with antibodies detecting the apoB component of Lp(a) or monoclonal antibodies to apo(a) directed to a unique epitope not present in kringle IV type 2.^22^ However, Lp(a) particles have been found to associate noncovalently with triglyceride-rich lipoproteins which could result in overestimation of Lp(a) measured by immunoassays based on the apo(a) capture/apoB detection approach.^22^,^29^ Another approach to circumvent the effect of apo(a) size variability, is the development of methods that are not antibody-based. Mass spectrometry methods have also been reported^30^ or are under development. However, to be of the greatest clinical utility, new assays should provide the same high throughput as the analytical platforms currently used in clinical labs as this will facilitate a more widespread measurement of Lp(a).

There is now an urgent need for the standardization of Lp(a) assays firstly, to meet growing demand with the recognition in international guidelines of this important, largely unmeasured cardiovascular risk factor and secondly, due to the prospect of novel treatments to dramatically lower Lp(a).^31^,^32^ Currently, lipoprotein apheresis is the only reliable means of achieving a substantial reduction of plasma Lp(a); however, its use is limited by its high cost, low capacity and lack of accessibility. Injectable antisense oligonucleotides targeting hepatic *LPA* RNA have been shown to reduce apo(a) production and apo(a) assembly with apoB, leading to >90% reduction in Lp(a) particle concentrations. These agents are now in Phase 3 trials (NCT04023552) and the results will confirm whether selective reduction of Lp(a) levels reduces CV risk. The need for clinical laboratories to deliver timely, accurate and standardized measurements of this enigmatic lipoprotein has become one of vital importance for us all to address.
